# Genetic Transformation of *Tribonema minus*, a Eukaryotic Filamentous Oleaginous Yellow-Green Alga

**DOI:** 10.3390/ijms21062106

**Published:** 2020-03-19

**Authors:** Yan Zhang, Hui Wang, Ruigang Yang, Lihao Wang, Guanpin Yang, Tianzhong Liu

**Affiliations:** 1 Key Laboratory of Biofuels, Key Laboratory of Shandong Energy Biological Genetic Resources, Qingdao Institute of Bioenergy and Bioprocess Technology, Chinese Academy of Sciences, Qingdao 266101, China; zhang_yan@qibebt.ac.cn (Y.Z.); wanglh@qibebt.ac.cn (L.W.); 2 University of Chinese Academy of Sciences, Beijing 100049, China; 3 College of Liberal arts and Sciences, National University of Defense Technology, Changsha 410073, China; h709966877@126.com; 4 College of Marine Life Sciences, Ocean University of China, Qingdao 266061, China; yguanpin@mail.ouc.edu.cn

**Keywords:** *Tribonema minus*, particle-gun bombardment, tubulin gene, green fluorescence protein, transformation

## Abstract

Eukaryotic filamentous yellow-green algae from the *Tribonema* genus are considered to be excellent candidates for biofuels and value-added products, owing to their ability to grow under autotrophic, mixotrophic, and heterotrophic conditions and synthesize large amounts of fatty acids, especially unsaturated fatty acids. To elucidate the molecular mechanism of fatty acids and/or establish the organism as a model strain, the development of genetic methods is important. Towards this goal, here, we constructed a genetic transformation method to introduce exogenous genes for the first time into the eukaryotic filamentous alga *Tribonema minus* via particle bombardment. In this study, we constructed pSimple-*tub*-*eGFP* and pEASY-*tub*-*nptⅡ* plasmids in which the green fluorescence protein (*eGFP*) gene and the neomycin phosphotransferase Ⅱ-encoding G418-resistant gene (*nptⅡ*) were flanked by the *T. minus*-derived tubulin gene (*tub*) promoter and terminator, respectively. The two plasmids were introduced into *T. minus* cells through particle-gun bombardment under various test conditions. By combining agar and liquid selecting methods to exclude the pseudotransformants under long-term antibiotic treatment, plasmids pSimple-*tub-eGFP* and pEASY-*tub- nptⅡ* were successfully transformed into the genome of *T. minus*, which was verified using green fluorescence detection and the polymerase chain reaction, respectively. These results suggest new possibilities for efficient genetic engineering of *T. minus* for future genetic improvement.

## 1. Introduction

Eukaryotic microalgae are a very diverse group of organisms that have been reported to play vital roles in the fixation of CO_2_, O_2_ production, and climate change [1,2]. Furthermore, many groups of microalgae have great potential to produce a variety of commercially valuable carbon compounds, including lipids, starch, and carbohydrates, and specifically, they are well known for their ability to produce long-chain fatty acids, such as polyunsaturated fatty acids [3,4,5]. Therefore, these microalgae with rapid growth rates have been considered as possible sources of next-generation energy fuels and high-value products.

The filamentous yellow-green algae *Tribonema*, which are widespread in freshwater and salt-water ecosystems, have gained substantial attention [6]. The *T. minus* strain has the ability to grow under autotrophic, mixotrophic, and heterotrophic conditions and synthesize large amounts of fatty acids, especially unsaturated fatty acids [7,8]. On one hand, *T. minus* is an industrial strain of microalgae that is used for biodiesel production under photoautotrophic conditions because of its high lipid content (up to 60.2% of its biomass) and easy harvesting characteristics due to its unbranched filaments. On the other hand, *T. minus* cells could be made economically feasible for the production of functional fatty acids with high value via heterotrophic/mixotrophic cultivation. In addition, a recent study by Wang et al. [9] revealed the molecular mechanisms of carbon partitioning during lipid accumulation in *T. minus* under photoautotrophic and heterotrophic conditions. Moreover, the recent genome sequencing of *T. minus* makes the species an attractive model for understanding the molecular nature of cell formation and lipid synthesis in the cells (the manuscript is undergoing preparation for publication). However, information on transgene expression and genetic engineering for understanding molecular mechanisms and strain development is very limited in the *Tribonema* genus. 

The availability of facile and efficient transformation techniques for transgene expression and metabolic engineering is important for biotechnological application. Specifically, species-specific molecular operations in microalgae will allow for the engineering of improved performances to drive down manufacturing costs and overcome the economic threshold for the production of commercially competitive products. The identification and genetic manipulation in this organism are crucial steps for the improvement of lipid productivity and/or the growth rate in microalgae, and the establishment of a genetic transformation method is the first step towards this goal. To date, suitable transformation techniques have been established only in several limited unicellular models of eukaryotic microalgae, such as *Chlamydomonas reinhardtii* [10,11], *Nannochloropsis* sp. [12,13], *Chlorella* [14], and *Phaedodactylum tricornutum* [15]. Unfortunately, no research group has reported the stable genetic transformation of filamentous eukaryotic microalgae, including those from the *Tribonema* genus.

In *Nannochloropsis* sp. and other green algae, electroporation is a useful tool to introduce exogenous DNA into cells partially deprived of their cell walls [16,17]. Therefore, in our previous study, considering the filamentous characteristic of *T. minus*, we first attempted to use electroporation to introduce the plasmid into the protoplasts of this alga. However, although we tried to degrade the *T. minus* cell wall using several different enzymes that weaken the cell walls of other microalgal cells [18,19], we have yet to prepare transformable protoplasts (data not shown). We then conducted particle bombardment experiments, as this method can successfully introduce exogenous DNA into cells with a rigid cell wall [20].

Here, in this study, the genetic transformation of *T. minus*, a natively robust and oleaginous algae, was investigated using a particle bombardment method. The efficient transformation conditions and some operational details in the transformation were determined by expressing the green fluorescent protein (*eGFP*) gene and neomycin phosphotransferase Ⅱ-encoding G418-resistant (*nptⅡ*) gene. These results suggest new possibilities for efficient genetic engineering of *T. minus* for future genetic improvements. This is the first report of a genetic engineering system in eukaryotic filamentous microalgae. 

## 2. Results 

### 2.1. Growth Characterization of T. minus in Response to Herbicides and Antibiotics

**Figure 1:**
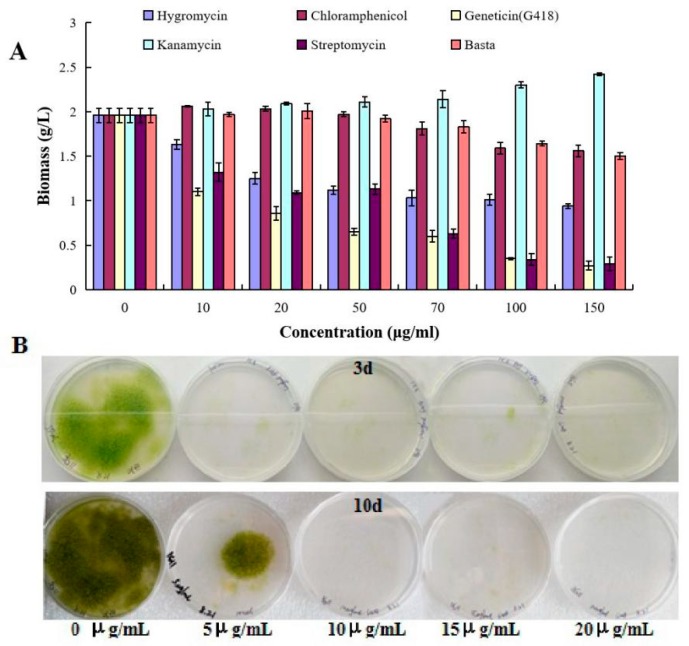
Analysis of herbicide and antibiotic sensitivities of *T. minus*. (**A**) Effects of different concentrations of all tested herbicides and antibiotics on the growth of *T. minus* in liquid medium. Five different antibiotics and one herbicide were used in this experiment, including hygromycin (light purple bars), chloramphenicol (dark red bars), geneticin (light yellow bars), kanamycin (light blue bars), streptomycin (dark purple bars), and basta (light red bars). Data are the average of *n* = 3 independent repeats; bars show SDs. Significant differences in biomass changes under each antibiotic test (*p* ≤ 0.05). (**B**) Growth of *T. minus* on the agar plates containing different concentrations of G418.

Transformability is usually tested using a plasmid harboring a selection marker (typically an antibiotic resistance gene) under the control of a promoter [21]. In order to select appropriate antibiotics for genetic manipulation, axenic cells of *T. minus* were first verified for herbicide and antibiotic sensitivity in liquid cultures. Five different antibiotics and one herbicide were assessed at various concentrations. The results showed that in liquid culture, *T. minus* is very sensitive to some of the antibiotics tested, particularly streptomycin and G418, causing 43.88% and 59.49% reductions in growth at concentrations of 10 and 20 μg mL^−1^, respectively, in 5 days (Figure 1A). *T. minus* was slightly less sensitive to hygromycin, which has been widely used as a selection marker for the transformation of eukaryotes [22,23], exhibiting growth reduction of about 50% at an antibiotic concentration of 50 μg mL^−1^. Chloramphenicol and basta did not have strong effects on the growth of *T. minus* at low concentrations, but higher concentrations (100 and 150 μg mL^−1^) caused considerable inhibition to the biomass concentration of *T. minus*. On the other hand, kanamycin had almost no effect on the growth of *T. minus* (Figure 1A).

Our tests in liquid medium allowed us to analyze the effects of antibiotics in a quantitative, repetitive, and reliable way. However, in order to provide further information about the concentrations of antibiotics, especially G418, that are appropriate for the selection of transformants, we also assessed the effects of G418 on agar plates. Subsequently, the sensitivity of *T. minus* to G418 for genetic selection was examined on BG11-based agar plates with different concentrations (5, 10, 15, and 20 μg mL^−1^) of G418. After 3 days, the cells cultured on agar plates without G418 had a good growth status, while those cultured with different concentrations of G418 did not show obvious growth. With prolongation of the cultivation, the results indicated that G418 at concentrations of 10 μg mL^−1^ and higher could effectively prevent the growth of *T. minus*, as the alga could still be grown on the plates at 10 days, giving a final concentration of 5 μg mL^−1^ (Figure 1B). Therefore, in accordance with the antibiotic sensitivity test, a G418-resistant gene encoding neomycin phosphotransferase Ⅱ (*nptⅡ)* was used as a selection marker gene [24] in this study. Additionally, the selective concentrations of 10 and 20 μg mL^−1^ were chosen for use in agar and liquid media, respectively. 

### 2.2. Construction of Plasmids pSimple-tub-eGFP and pEASY-tub- nptⅡ

**Figure 2:**
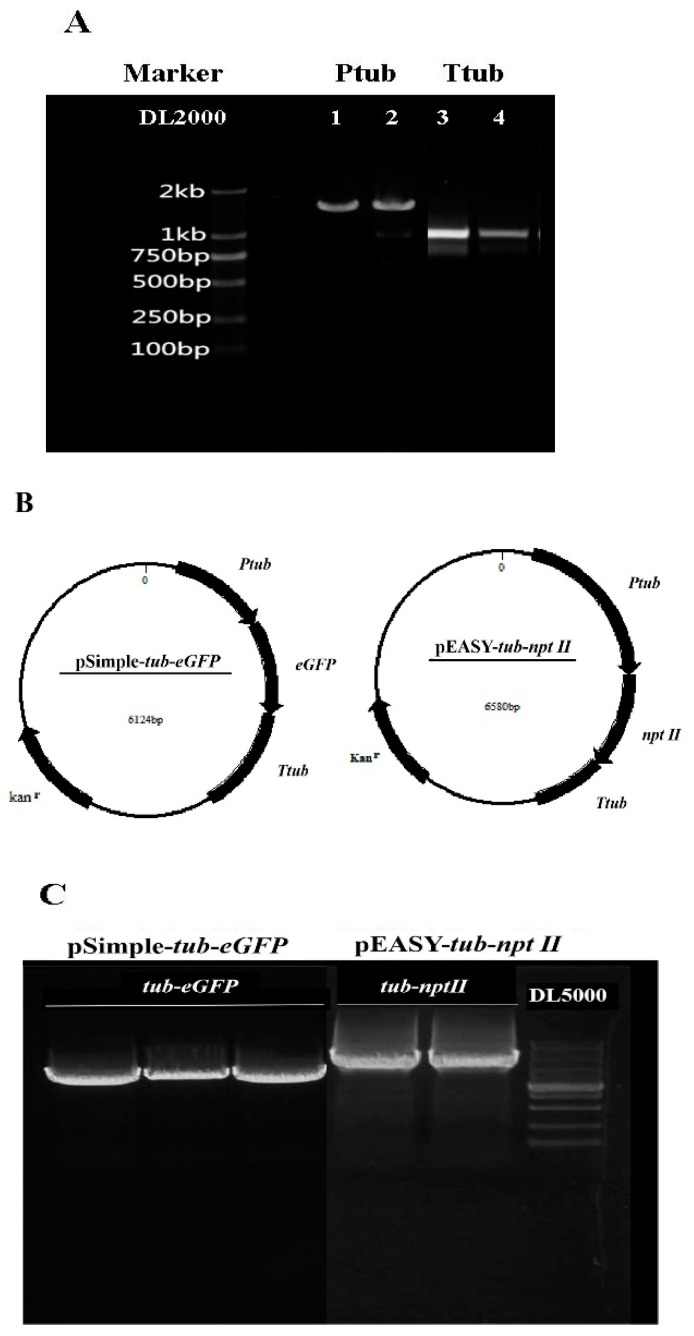
Construction of pSimple-*tub-eGFP* and pEASY-*tub-nptⅡ* vectors. (**A**) Gel analysis of *tub* promoter and terminator. (**B**) Design structure and size of pSimple-*tub-eGFP* and pEASY-*tub-nptⅡ* vectors. (**C**) Gel analysis of pSimple-*tub-eGFP* and pEASY-*tub-nptⅡ* vectors.

Besides the resistance selection marker, promoters and terminators are the other important elements for plasmid construction that direct transcription and termination, respectively. From the transcriptomic data, we found that the tub gene is expressed constitutively at a high level in *T. minus*, and thus, we used the upstream and downstream regions of the tub gene to provide promoter and terminator functions, respectively. We cloned and sequenced the tub gene (annotation number of MN481481 in Genbank) and its flanking sequences by the gene walking method, as described in the Methods section, and finally, we found that the nucleotide sequences comprised a promoter region (1373bp) and a terminator region (574bp) from the tub gene in *T. minus* (Figure 2A).

A fusion PCR method was performed to fuse the genes of *eGFP* and *nptⅡ* with the tub promoter and terminator of *T. minus*. After three fusions, we constructed two vectors that were controlled by the endogenous tub promoter and terminator with sizes of 6124 and 6580bp, respectively, and we named the vectors pSimple-*tub-eGFP* and pEASY-*tub-nptⅡ* (Figure 2C). The sequences of pSimple-*tub-eGFP* and Peasy-*tub-nptⅡ* were also submitted to Genbank with the accession numbers MN496120 and MN481464, respectively.

### 2.3. pEASY-Tub-nptⅡ Transformant Selection

In a previous study, it was suggested that M17 tungsten particles work as DNA carriers because gold particles can easily adhere to the inner wall of Eppendorf tubes, which might lead to difficulties in the DNA coating process [25]. Additionally, the particles coated with at least 1 μg DNA were utilized for one bombardment. Here, plasmid pSimple-*tub-eGFP* and pEASY-*tub-nptⅡ* were transferred into *T. minus* cells by particle bombardment with various pressure conditions, as listed in the Methods section. In former tests, *T. minus* cells were easily shattered as the tungsten powder appeared on the filter membrane under higher pressure conditions. Hence, we layered another piece of filter membrane on the upper part of the cells prepared for transformation under a pressure of 900 psi and higher.

**Figure 3:**
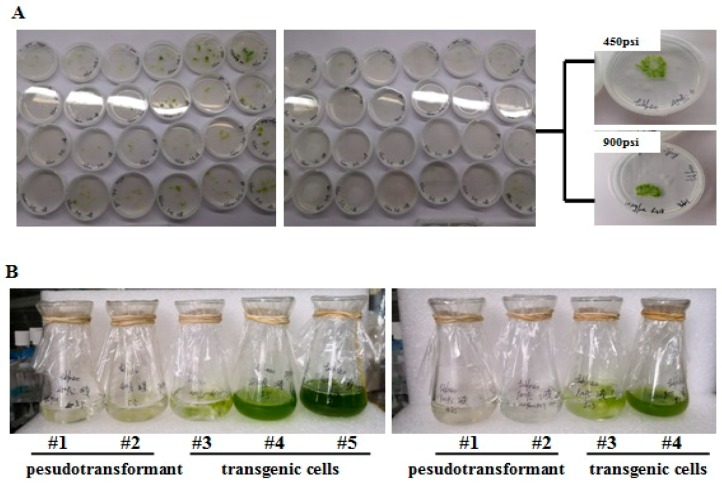
Agar and liquid selecting of transgenic cells. (**A**) Cells grown on an agar plate containing 10 μg mL^−1^ G418. (**B**) After growth, cells were transferred from the agar plate to BG11 medium supplemented with 20 μg mL^−1^ G418.

For pEASY-*tub-nptⅡ* transformants, after regeneration, cells were transferred onto an agar plate containing 10 μg mL^−1^ of G418. About 10 days later, we observed obvious vigorous congregated cells in agar that transformed under two pressure conditions of 450 and 900 psi (Figure 3A); however, no cells survived or were transferred under higher pressure levels, as the cells went grey. What needs to be emphasized is that *T. minus* cells on the agar plates exist in the form of an aggregate, not a single colony, because of their filaments. 

Nevertheless, there might be such a phenomenon where there was no cell that transformed into exogenous DNA in the filament, but we could still observe the cells growing on the plates because there were some cells that did not directly contact antibiotics due to the filamentous characteristic. That resulted in some pseudotransformants. Therefore, we tried our best to pick as few cells as possible to be transferred into BG11 liquid medium supplemented with 20 μg mL^−1^ G418 for further screening of transgenic cells, because the transformant cells could produce many new transgenic cells due to the genus growing by cell division [26], while the cells that did not transform successfully died under long-term antibiotic treatment. Figure 3B shows the growth status of cells transferred from G418-resistance agar plates. This revealed that regardless of the pressure, 450 psi or 900 psi, not all cells that survived in the agar plates could grow under long-term antibiotic treatment in BG11 liquid medium.. It could be suggested and concluded that the transgenic cells grew on the selection, whereas the wild-type and pseudotransformant cells were not able to survive due to selection pressure. 

### 2.4. Confirmation of Transformation into the T. minus Genome

**Figure 4:**
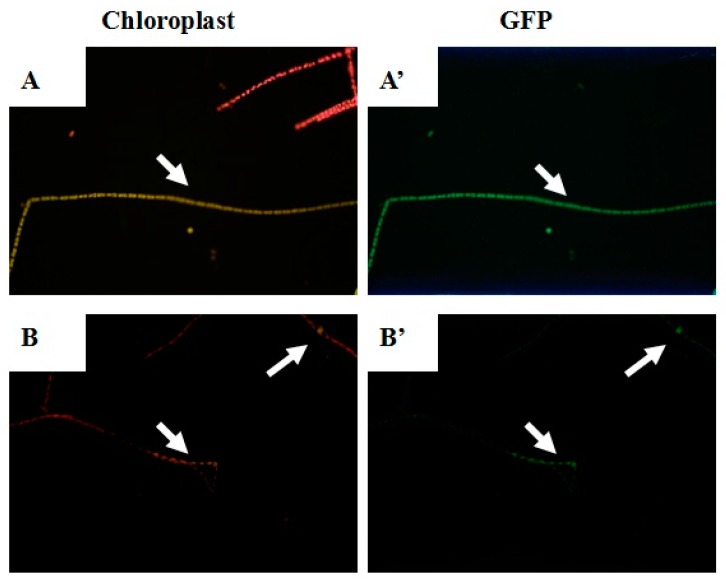
Phenotypic characterization of pSimple-*tub-eGFP* transformed *T. minus* under 450 psi. Detection was in the range of 685–735 nm for chloroplast autofluorescence (**A**, **B**) and 495–556 nm for *eGFP* (**A’**, **B’**).

After 48 h of regeneration, pSimple-*tub-eGFP* transformants were immediately observed with fluorescence analysis. Since the fluorescence from GFP was green and the autofluorescence from chloroplasts was red, transformant cells could be easily distinguished from wild-type cells. We observed the cells treated with particle bombardment under 450, 900, 1100, and 1350 psi; however, there was almost no obvious corresponding color found in samples of 900, 1100, and 1350 psi. Phenotypic characterization of *T. minus* transformed by pSimple-*tub-eGFP* under 450 psi is illustrated in Figure 4. The red autofluorescence from chloroplasts in wild-type cells is shown in Figure 4A,B, while green fluorescence was observed in transformed cells containing the *eGFP* gene. Figure 4 gives intuitive evidence that the *eGFP* gene was successfully transformed into *T. minus* cells under 450 psi. Interestingly, the color of the entire filament changed, as shown in Figure 4A,A’; however, as shown in Figure 4B,B’, the corresponding color was just observed in single or several cells. This is a normal phenomenon because it is difficult for the *eGFP* genes to be transformed into each cell composing the entire filament. 

**Figure 5:**
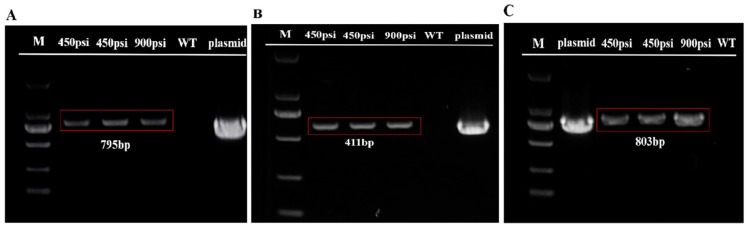
PCR amplification from genes using the genomic DNA of *T. minus* transformants and wild types (marker: DL2000). Gel analysis of the *nptⅡ* (**A**) and *eGFP* genes (**B**) in transformants and wild types. (**C**) Gel analysis of fragment in pEASY-*tub-nptⅡ* in third-generation transformants and wild types.

In order to check the presence of the plasmid DNA in transformants, both the predicted transgenic cells that grew well in BG11 medium supplemented with 20 μg mL^−1^ G418 and the pSimple-*tub-eGFP* positive transformants were examined by genomic PCR analysis. Transgenic cells under 450 and 900 psi were picked, and the genomic DNA was extracted and used for PCR amplification of the *nptⅡ* and *eGFP* gene fragments, respectively. As illustrated in Figure 5A,B, both the *nptⅡ* (795bp) and *eGFP* genes (411bp) were detected in transformants. Moreover, 18S rDNA was detectable in all samples except for the vector control, indicating the successful transformation of *T. minus* (data not shown). 

In addition, three pEASY-*tub-nptⅡ* transgenic cells under 450 and 900 psi were picked randomly and transferred into fresh BG11 medium for a subculture until the third generation. Moreover, according to the sequence of pEASY-*tub-nptⅡ,* we chose a middle fragment with a size of 803 bp including part of the *nptⅡ* gene and part of the tub promoter at random and designed the primers to have sequences of T-PK-F (5’-gcaatttgattggccagcgcaac-3’) and T-PK-R (5’-gatggatactttctcggcaggag-3’). We amplified this fragment in the genomic DNA extracted in the third-generation transgenic cells, and the wild type was regarded as the control. Not surprisingly, Figure 5C reveals the presence of this fragment in all transgenic cells. 

### 2.5. Quantitative Analysis of the nptⅡ Gene in the Transformants

**Figure 6:**
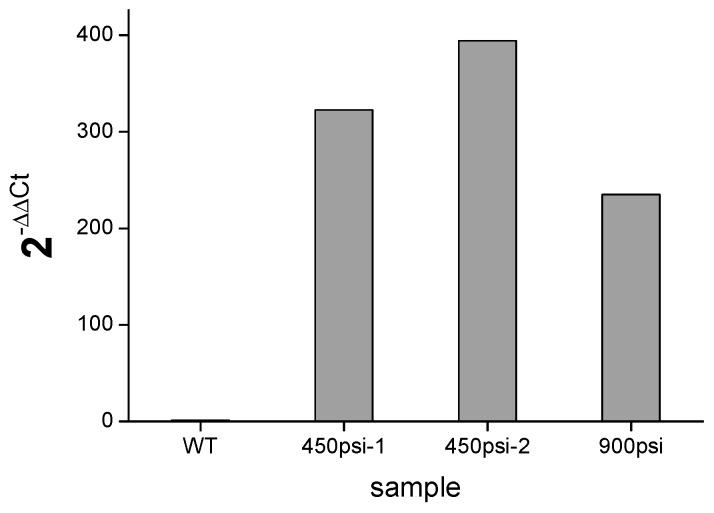
Quantification of the *nptⅡ* gene in three transformants of *T. minus* through real-time PCR.

Quantitative real-time PCR was performed to quantify the efficiency of genomic integration and transcription of the introduced *nptⅡ* genes in *T. minus* transformants. Figure 6 shows their quantitative results in the cDNA obtained from three independent transgenic cells of *T. minus* bombarded with pEASY-*tub-nptⅡ* under 450 and 900 psi, respectively. The endogenous *tub* gene was utilized as a reference gene, and the samples from wild-type *T. minus* were used as the control. Normalized fold changes of the *nptⅡ* gene in these transformants to the wild-type cells were calculated with the *tub* gene as a balance based on the ΔΔCt method. The signals of *nptⅡ* genes identified in the transformant genomes were calculated to be 235–394 times higher than those in the wild-type cDNA. All these results confirm that the plasmids were successfully integrated into the genome and expressed efficiently in *T. minus* transformants.

## 3. Discussion

*Tribonema minus*, which is classified within the family Xanthophyceae, has recently become an attractive organism for use in biotechnology. This alga has the ability to grow under autotrophic, mixotrophic, and heterotrophic conditions, and it can synthesize and accumulate a significant amount of fatty acid intracellularly [27]. Establishing genetic tools for *T. minus* would increase the potential value of this alga in biotechnological applications, and it could serve as a molecular model for Xanthophyceae. 

Transformability is usually tested using a selection marker; thus, in order to develop a system for the efficient selection of transformants, knowledge about the sensitivity of a strain to various antibiotics is essential. Selectable marker genes, including neomycin phosphotransferaseⅡ (*nptⅡ*) [24], phleomycin-resistance (*shble*) [25], and streptothricin acetyltransferase (*sat*) [21], have been used for microalgae transformation. According to an antibiotics sensitivity trial, *T. minus* is very sensitive to G418 (neomycin); therefore, the resistance gene *nptⅡ* was used in this study as the selection maker. Active promoters and terminators are important constitutions of efficient transformation and expression vectors. Researchers once chose heterologous eukaryotic vectors, such as SV 40 [28] or CaMV 35S promoters [29], to drive foreign genes into microalgae cells; however, results showing successful transformation could not be repeated widely [30]. Generally, the utilization of endogenous vectors could greatly facilitate the integration and expression of foreign genes in the host genome [23]. Alpha tubulin (*tub*) is a principal component of microtubules that forms part of the cytoskeleton in eukaryotic cells [31], and its promoter and terminator have been widely utilized for the construction of efficient screening markers in different organisms [25]. Transcriptomic data show that the *tub* gene has a constitutive and high level of expression in *T. minus*. Thus, the upstream and downstream regions of endogenous *tub* could be considered to provide active promoter and terminator functions. In this work, the complete sequence of the *tub* gene, including its exons, introns, and 5’ and 3’ flanking sequences were successfully identified in *T. minus*. Subsequently, a fusion PCR method was performed to fuse the genes of *eGFP* and *nptⅡ* with the tub promoter (1373 bp) and terminator (574bp) of *T. minus*, respectively. Finally, two vectors that were controlled by the endogenous tub promoter and terminator with sizes of 6124 and 6580 bp were successfully constructed and named pSimple-*tub-eGFP* and pEASY-*tub-nptⅡ*, respectively. 

During biolistic transformation manipulation, many manipulation details influenced the success rate [32]. The culture phase and growth status of microalgae cells are very important for bombardments. When a late-stage culture with predominantly *H. pluvialis* cells was used for bombardment, no successful transformation was achieved. However, the transient expression of a gene could be served in a logarithmic culture with cells after bombardment [25,28]. In this work, we also chose the cells at the logarithmic phase and gave a short incubation period for cells on a solid medium plate. Moreover, the transformation of microalgae cells could also be influenced by the pressure of bombardment. In our former tests, *T. minus* cells were easily shattered as the tungsten powder appeared on the filter membrane under higher pressure conditions. Hence, we layered a piece of filter membrane on the upper part of the cells prepared for transformation under a pressure of 900 psi and higher. Transformation was achieved successfully under only two lower pressure conditions of 450 and 900 psi. The effective pressures were lower than those used in the genetic transformation of a unicellular green microalgae *Pseudochoricystis ellipsoidea*, the bombardment of which can achieve success from 900 to 1550 psi [33]. This might be related to the loose degree of filaments and gives fundamental manipulation parameters for the bombardment of filamentous microalgae. 

Different from unicellular microalgae, the transformant screening of filamentous *T. minus* was carried out on agar plates of G418-containing medium followed by further resistant treatment in liquid medium. For unicellular microalgae, the transformant colonies could be easily observed on the agar plates [34,35]; however, it was difficult to grow a colony of *T. minus* transformants instead of a flake or a speck of green cells on the agar plates (Figure 3A). A possible explanation is that although some single cells composed the filaments, they were not transformed into exogenous DNA, it was possible that they did not contact antibiotics directly due to their filamentous characteristics. However, the single transformant cells could give many new transgene cells due to the genus growing by cell division [26], but those cells in the same filament that did not transform successfully would die under long-term antibiotic treatment. Therefore, after agar-plate screening, we picked as few survival cells as possible and transferred them into liquid medium with a higher G418 concentration. After a period of resistant treatment, some cells showed a good growth status in liquid medium, while some died. The *nptⅡ* fragment was amplified in the cells that grew well in antibiotic-containing liquid medium, which proved the success of transformation and resistant screening. Moreover, the results of the RT-PCR analysis confirmed that the plasmid was successfully integrated into the genome and expressed efficiently in *T. minus* transformants. Furthermore, to detect the stability of transformation, we subcultured the transformant and examined the partial vector fragment with a size of 803 bp, including part of the *nptⅡ* gene and part of the tub promoter at random. The final result clearly indicated that *nptⅡ* was still in the transformant cells, at least in the third generation. 

In conclusion, stable nuclear transformation was achieved for the first time in *T. minus* through biolistic bombardment using the *nptⅡ* gene driven by transcriptional elements flanking the endogenous *tub* gene. This approach enriched the molecular tools for the genetic modification of *T. minus*, and a transformant screening flow was well established. On the basis of this, further research into the metabolic engineering and synthetic biology of *T. minus* should be done. 

## 4. Materials and Methods 

### 4.1. Strain and Growth Conditions

The yellow-green microalgae *Tribonema minus* SAG 880-3 was obtained from the Culture Collection of Algae of Gottingen University and maintained in our laboratory. *T. minus* cells were grown at 23 ± 1 ℃ under continuous fluorescent light (100 μmol m^−2^s^−1^) in liquid BG11 medium [36] that was continuously bubbled with air containing 1% (v/v) CO_2_. The *Escherichia coli* strain DH5α was used for the construction, propagation, and maintenance of plasmid. It was cultured in Luria–Bertani (LB) medium at 37 ℃. 

### 4.2. Sensitivity to Herbicide and Antibiotics

The effects of the herbicide basta (Sigma) and the antibiotics hygromycin, chloramphenicol, geneticin (G418), kanamycin, and streptomycin (Sigma) on *T. minus* in liquid BG11 medium were monitored. Aliquots of BG11 medium supplemented with varying concentrations of basta or each antibiotic (10, 20, 50, 70, 100, or 150 μg mL^−1^) were inoculated with freshly grown *T. minus* to an initial biomass concentration of 0.2 g L^−1^ (OD_680_ and density may not be accurate because of the filaments). 

To further determine the sensitivity of *T. minus* to G418 on solid BG11 medium, aliquots of 200 μL suspension (0.2 g L^−1^) were inoculated on agar plates containing different concentrations of G418 (5, 10, 15, and 20 μg mL^−1^). The flasks and agar plates were incubated under continuous light at an irradiance level of 60 μmol m^−2^s^−1^ and 23 ± 1 °C, and the growth of *T. minus* in the liquid and the solid medium was monitored at daily intervals for 10 days and 2 weeks, respectively. The sensitivity of *T. minus* to the antibiotics and the herbicide was repeated at least three times. 

### 4.3. Endogenous Promoter and Terminator Clone

A homology search using BLASTX was performed with reference to the 13,751 genes from the transcriptomic sequence of *T. minus* to obtain *tub* genes. Through PCR with designed primers (Table 1), the *tubulin* (*tub*) gene was obtained and the nucleotide sequences comprised a promoter region and a terminator region from a *tub* gene in *T. minus*. Genome walking [37] with a Genome Walking Kit (TaKaRa Bio Inc., Kusatsu, Japan) was used to obtain the 3’ flanking sequences of the *tub* gene. Three specific synthetic primers, tub-sp1, tub-sp2, and tub-sp3 (Table 1), were designed. Each primer had a relatively high annealing temperature. Three rounds of PCR were performed for each walking process using the product of the previous PCR as a template for the next PCR. Each PCR mixture had 1×LA PCR Buffer Ⅱ (Mg2+ plus) containing 0.4 mM dNTP mixture, 2.55U TaKaRa LA Taq, 0.2μM of each primer, and a certain amount of template. The first round of PCR had three annealing stages: stage 1 had five high-stringency (65 ℃) cycles; stage 2 had one low-stringency (25 ℃) cycle; and stage 3 had thirty high-stringency (65 ℃) cycles. The next two rounds of PCR had two annealing stages: stage 1 had 30 high-stringency (65 ℃) cycles and stage 2 had 15 low-stringency (44 ℃) cycles. After agarose gel electrophoresis and gel extraction, the fragments were cloned into pEASY-T1 vectors and sequenced.

### 4.4. Construction of Plasmids for Transformation

For the transformation, we constructed two vectors, the pSimple-*tub-eGFP* and pEASY-*tub-nptⅡ* plasmids, in which the green fluorescent protein (*eGFP*) gene [38] and the neomycin phosphotransferase Ⅱ-encoding G418-resistant gene (*nptⅡ*) were flanked by the *T. minus*-derived tub promoter (Ptub) and terminator (Ttub), respectively. The A fusion PCR method was performed to fuse the *nptⅡ* and *eGFP* genes with the *tub* promoter and terminator of *T. minus,* as described in a previous study [25]. Taking the construction of the tub/*nptⅡ* cassette as an example, three target fragments, Ptub, *nptⅡ*, and Ttub, were obtained by regular PCR with the primer pairs of Ptub-F/Ptub(*nptⅡ*)-R, nptⅡ-F/ *nptⅡ*-R, and Ttub(*nptⅡ*)-F/Ttub-R, respectively (Table 2). The primers for pSimple-*tub-eGFP* are listed in Table 3. The three purified DNA fragments in equal mol amounts were mixed with *pfu* DNA polymerase in a 50 μL PCR reaction system. This mixture underwent a PCR fusion procedure as follows: a 10 min initial denaturation step at 95 ℃ followed by thirteen cycles of denaturation for 30 s at 95 ℃, annealing for 30 s at 55 ℃, extension for 2–3 min at 72 ℃, and a final 10 min extension step at 72 ℃. The final mixture was utilized as the template for PCR amplification of the target tub/*nptⅡ* fragment with the primer pair of tub-f0/tub-r0. The resulting fusion fragments were cloned into the plasmid pEASY-T1. The verified plasmids were named pEASY-*tub- nptⅡ*. 

### 4.5. Particle Bombardment

For transformation, *T. minus* cultures at the logarithmic growth phase were pelleted by filtration and suspended in BG11 medium to a biomass concentration of 2–3 g L^−1^. Then, a 2 mL aliquot of cell suspension was layered directly onto the central area of an MF-Millipore membrane filter (Millipore, Bedford, MA, USA), which was placed on a BG11 solid plate. After growth for 2 days, the samples were used as the recipient cells for transformation.

The bombardment was performed with a PDS-1000/He Biolistic Particle Delivery system (Bio-Rad, USA). Plates were bombarded from a distance of 6 cm and bombarded under vacuum pressure at 125 mm Hg using helium pressures of 450, 900, 1100, and 1350 psi to accelerate particles. DNA coated particles were prepared by mixing 50 μL of 17 tungsten particle solution (60 mg mL^−1^ in H_2_O) with 5 μL of a DNA solution (>1 μg μL^−1^), 50 μL of 2.5 M CaCl_2_, and 20 μL of 0.1M spermidine base. This was followed by 10 min incubation on ice and two centrifugal washes with 70% and then 100% ethanol before final resuspension in about 50 μl ethanol. A total of 10 μL of prepared DNA-coated particle solution was layered on a microcarrier for one trial. 

After transformation, cells on the membrane were transferred into 10 mL of fresh BG11 medium and incubated at 23 ± 1 °C under dark conditions for 2 days for regeneration. Then, 200 μL of culture transformed with pEASY-tub-*nptⅡ* were plated onto BG11 agar plates containing 10 μg mL^−1^ G418. After 2–3 weeks, the cells grown on the plates were picked up and resuspended in 20 mL of BG11 medium supplemented with 20 μg mL^−1^ G418 and incubated under continuous fluorescent light with 60 μmol photons m^−1^s^−1^ at 23 ± 1 ℃ for further transgenic cell screening. 

### 4.6. Fluorescence Detection of eGFP Gene Expression

pSimple-*tub-eGFP* transformants grown in medium were used for the fluorescence analysis along with wild-type *T. minus* cells as a negative control. Leica DM14000 (Leica Microsystems, Germany) was used to examine *eGFP* expression using excitation at 488 nm. Detection was in the range of 495–556 nm for *eGFP* and 685–735 nm for chloroplast autofluorescence.

### 4.7. Preparation of Genomic DNA and PCR Analysis

The G418-resistant *T. minus* cells were disrupted by grinding in liquid nitrogen with a mortar and pestle and transferred to a 1.5 mL microcentrifuge tube. The cells were suspended in TEN buffer (10 mM Tris-HCl, pH 8.0, 150 mM NaCl, 10 mM EDTA). After phenol/chloroform/isoamylalcohol extraction and 2-propanol precipitation, samples were treated with RNase A, and the genomic DNA was recovered by ethanol precipitation following phenol/chloroform/isoamyl alcohol and chloroform treatments. 

Genomic PCR was performed with isolated 0.1 μg genomic DNA as a template and 5 μL of Go Taq Master Mix. Amplifications were performed by incubating reaction mixtures at 95 ℃ followed by 20 s at 52.5 ℃ and 60 s at 72 ℃. Assays with no template were examined for every experiment as a negative control.

### 4.8. Continuous Cultivation of Transformants 

The G418-resistant *T. minus* transformants were harvested via filtration. After being washed with distilled water twice, the cells were transferred into fresh BG11 medium for subculture. The extracted genomic DNA and PCR analysis of the resistant gene from transformants, which was cultured to the third generation, were examined and analyzed as described above.

### 4.9. Quantitative Real-Time PCR Analysis

Total RNA was prepared by the ConcertTM Plant RNA Reagent (Invitrogen, Carlsbad, CA, USA) according to the manufacturer’s instructions and purified with an RNeasy Mini Kit column (QIAGEN, Hilden, Germany). The purified RNA (1.5 μg) was transcribed into cDNA by employing the Transcriptor High Fidelity cDNA Synthesis Kit (Roche Applied Science, Mannheim, Germany), as described by the manufacturer. PCR was performed as for the genomic PCR analysis described above with slight modifications. The conditions of the PCR were as follows: 95 ℃ for 2 min prior to 25 cycles of 15 s at 95 ℃, followed by 20 s at 52.5 ℃ and 60 s at 72 ℃. Assays with samples that were prepared without reverse transcriptase in the cDNA synthesis reaction and with no template were examined for every experiment as a negative control.

## Figures and Tables

**Table 1:** Primers prepared for the *tub* promoter and terminator via genome walking.

Primers	Sequence (5’-3’)
tub-F	ATGCGTGAATGCATCAGCATCC
tub-R	CGGCACACGTCGTACAGGGCC
Ptub-sp1	AGTAGAGCTCCCAGCAGGCATT
Ptub-sp2	TTTCAGGGCATCGCTGCTTCAGTA
Ptub-sp3	AGCGGAACTCATGCCGATCAGGTA
Ttub-sp1	TAACCCCACTCCTCCTCGTCGCTTT
Ttub-sp2	ATCAACTACCAGCCTCCCACCGT
Ttub-sp3	ACCACAAGTTCGACCTCATGTACGCC

**Table 2:** Primers prepared for construction of pEASY-*tub- nptⅡ* via fusion PCR.

Primers	Sequence (5’–3’)
Ptub-F	CAGTGCAGTCATCTGCTGCGGGC
Ptub(*nptⅡ*)-R	GCAATCCATCTTGTTCAATCATGTTGACTGGAGAAGTGGTCCTGC
*nptⅡ*-F	GCAGGACCACTTCTCCAGTCAACATGATTGAACAAGATGGATTGC
*nptⅡ*-R	GTGCACACGTCAACAGTGCGCTCAGAAGAACTCGTCAAGAAGG
Ttub(*nptⅡ*)-F	CCTTCTTGACGAGTTCTTCTGAGCGCATGTTGACGTGTGCAC
Ttub-R	GGGCGATTGGGCCCTGTAGATGC

**Table 3:** Primers prepared for the construction of pSimple-*tub-eGFP* via fusion PCR.

Primers	Sequence (5’–3’)
Ptub-F	GAATTCTCATGGCGCGACGCTGT
Ptub(eGFP)-R	GCTCCTCGCCCTTGCTCACCATGGTGGCCTTTAAGGGTGCTGTTTAAACTGC
eGFP-F	GCAGTTTAAACAGCACCCTTAAAGGCCACCATGGTGAGCAAGGGCGAGGAGC
eGFP-R	GTGGGCCTGAGCGTCATGTTTACTTGTACAGCTCGTCCATG
Ttub(eGFP)-F	CATGGACGAGCTGTACAAGTAAACATGACGCTCAGGCCCAC
Ttub-R	GGTTCATCTGCTGACGAGTGCT
